# Experimental determination of the steady-state charging probabilities and particle size conservation in non-radioactive and radioactive bipolar aerosol chargers in the size range of 5–40 nm

**DOI:** 10.1007/s11051-015-2981-x

**Published:** 2015-04-05

**Authors:** Peter Kallinger, Wladyslaw W. Szymanski

**Affiliations:** Faculty of Physics, University of Vienna, Boltzmanngasse 5, 1090 Vienna, Austria

**Keywords:** Aerosol, Nanoparticles, Bipolar diffusion charging, Soft X-ray, AC-corona discharge, Radioactive charger

## Abstract

Three bipolar aerosol chargers, an AC-corona (Electrical Ionizer 1090, MSP Corp.), a soft X-ray (Advanced Aerosol Neutralizer 3087, TSI Inc.), and an α-radiation-based ^241^Am charger (tapcon & analysesysteme), were investigated on their charging performance of airborne nanoparticles. The charging probabilities for negatively and positively charged particles and the particle size conservation were measured in the diameter range of 5–40 nm using sucrose nanoparticles. Chargers were operated under various flow conditions in the range of 0.6–5.0 liters per minute. For particular experimental conditions, some deviations from the chosen theoretical model were found for all chargers. For very small particle sizes, the AC-corona charger showed particle losses at low flow rates and did not reach steady-state charge equilibrium at high flow rates. However, for all chargers, operating conditions were identified where the bipolar charge equilibrium was achieved. Practically, excellent particle size conservation was found for all three chargers.

**Electronic supplementary material:**

The online version of this article (doi:10.1007/s11051-015-2981-x) contains supplementary material, which is available to authorized users.

## Introduction

Almost every technique for the measurement and manipulation of aerosol nanoparticles utilizes electrostatics which makes the charging of nanoparticles an essential requirement for the use of such methods. Probably, the most common application of a charger in the field of aerosol science is the use in combination with a DMA. For this purpose, usually a bipolar diffusion charger is used. In most cases a radioactive source (e.g. ^241^Am, ^210^Po, ^85^Kr) is utilized to create bipolar ions from air inside the charger. The advantages of this method are the ease of use and the well-defined charge distribution. However, the safety risk and very strict legal regulations concerning the use of radioactive material are making this method increasingly unpopular and have induced a still ongoing search for suitable substitutes.

The diffusion charging mechanism relies on the collision of air ions with aerosol particles due to Brownian motion and electrostatic forces. It can either be unipolar (when air ions of only one polarity are present) or bipolar. While unipolar diffusion charging typically has a higher charging efficiency than bipolar diffusion charging it produces also a higher amount of multiply charged particles and does not deliver a very reproducible charge distribution. This makes it less attractive for the use in combination with a DMA (e.g. Laschober et al. 2006). In a bipolar diffusion charger a steady-state charge equilibrium establishes after a certain time as a result of the competition of ions of both polarities which are continuously charging and discharging the particles in both polarities. This charge equilibrium is independent of the initial charge distribution of the particles entering the charger (Fuchs 1963).

Measurements done with radioactive chargers (e.g. Hussin et al. 1983; Kousaka et al. 1983; Reischl et al. 1983; Adachi et al. 1985; Wiedensohler et al. 1986; Wiedensohler and Fissan 1991; Reischl et al. 1996; Alonso et al. 1997; Covert et al. 1997) have shown that the charging model by Fuchs (1963) with a small correction of the ion–aerosol collision probability (Hoppel and Frick 1986) fits best with experimental data. However, one should keep in mind that the Fuchs model depends on several input parameters like the ion mobilities and ion masses which were often slightly adjusted to achieve a good agreement between the theory and the measurements.

Instead by means of a radioactive source, the air ions required for the diffusion charging process can also be produced by e.g. soft X-ray irradiation or an electrical discharge like corona discharge. X-ray irradiation has already been used to charge aerosol particles since more than a century (e.g. Millikan 1913), but it was Shimada et al. (2002) who reintroduced this method in recent years to our knowledge at first for aerosol particle measurements. While a soft X-ray charger still utilizes harmful radiation which has to be shielded, it has the advantage that it can be switched on and off. However, soft X-ray as well as radioactivity-based chargers have a potential for usually unwanted radiolytic particle production (Leong et al. 1983; Yun et al. 2009; Kallinger 2010). This effect can be brought under control by using higher flow rates (shorter residence time of the aerosol in the charger), the introduction of a radical scavenger, or as in the case of the soft X-ray charger used in this study, by including an attenuating window to reduce the intensity of the soft X-ray irradiation (Kaufman 2010). Several studies have shown a comparable charging performance of a soft X-ray charger in direct comparison with a radioactive ^241^Am-charger (Shimada et al. 2002; Lee et al. 2005; Yun et al. 2009; Kallinger et al. 2012). However, with exception of a few very recent publications (Jiang et al. 2014; Yoon et al. 2015; He and Dhaniyala 2014), there is still a lack of accurate data on the charged fractions of soft X-ray chargers, especially in the size range we investigated.

A direct current (DC) corona discharge produces only unipolar ions for the charging of aerosol particles. To create a bipolar ion atmosphere either two corona discharges with different polarities can be used (e.g. Adachi et al. 1993; Romay et al. 1994; Qi and Kulkarni 2013) or an alternating current (AC) voltage can be applied on a single active electrode producing positive and negative ions alternately (Zamorani and Ottobrini 1978; Stommel and Riebel 2004). Since the ions in such chargers are produced by two different processes (Goldman et al. 1985), the ion concentration ratio (i.e. ratio of the concentration of positive and negative ions) has to be controlled in order to produce a predictable charge distribution. The high strength of the electric field of a corona discharge can lead to particle losses if the discharge takes place directly inside the aerosol chamber. Therefore, corona chargers are often designed in a way that the aerosol does not come in contact with the electric field. Furthermore, particle production can also be an issue of corona-based chargers due to sputtering and gas to particle conversion (Romay et al. 1994). To the best of our knowledge, experimentally determined charging probabilities of aerosol nanoparticles in an AC-corona charger have not yet been reported in the literature.

In this study, we report the experimental investigation of two non-radioactive bipolar chargers based on soft X-ray irradiation and AC-corona discharge for the charging of aerosol nanoparticles in the size range of 5–40 nm. The charged particle fractions of both polarities and the particle size conservation (i.e. particle size is conserved if the charging process has no effect on the measured particle size) were measured by means of a tandem DMA technique. The measurements were done with different aerosol flow rates through the charger in the range of 0.6–5.0 liters per minute (lpm). For comparison, a radioactivity-based ^241^Am-charger was also investigated under the same conditions.

## Experimental

### Investigated chargers

#### Electrical ionizer

The “Electrical Ionizer” (EI) (Model 1090, MSP Corp., Shoreview, MN, USA) creates bipolar ions by means of an AC-corona discharge (with one corona needle). The manufacturer’s specification suggests the usable flow rates from 0.5 to 5.0 lpm and the aerosol particle diameter range from 10 nm to 10 µm (MSP Corp. 2010).

#### Advanced aerosol neutralizer

The “Advanced Aerosol Neutralizer” (AAN) (Model 3087, TSI Inc., Shoreview, MN, USA) is based on soft X-ray irradiation (photon energy <9.5 keV). The aerosol flow rate rage range is 0.3–5.0 lpm. A submicron aerosol size range is specified by the manufacturer (TSI Inc. 2012). For schematic drawings of the AAN and the EI, please refer to Kallinger et al. (2012). A more detailed drawing of the AAN can also be found in the supplemental information of Jiang et al. (2014).

#### ^241^Am-charger

For comparison, a radioactive charger (tapcon & analysesysteme, Salzburg, Austria) was used containing an ^241^Am foil with an activity of 60 MBq (1.6 mCi) (Steiner and Reischl 2012).

The volumes of the charging zones of the EI, AAN and ^241^Am-charger are approximately 30, 170, and 30 cm^3^, respectively.

During the measurements, the non-radioactive chargers were switched on and off. Since a radioactive charger cannot be turned off, a parallel arrangement of the charger and an identically built dummy (without a radioactive source) was used. The aerosol stream was split upstream of the charger and dummy and combined downstream afterwards. A pinchcock was used downstream of charger/dummy to switch between the two stream lines. In this manuscript, the ^241^Am-charger will be called “on” when the aerosol was routed through the charger and “off” when the aerosol was routed through the dummy. For the non-radioactive chargers, the words “on” and “off” will refer to the status of the ionizing source.

### Charging performance and particle size conservation

#### Measurement principle

**Fig. 1:**
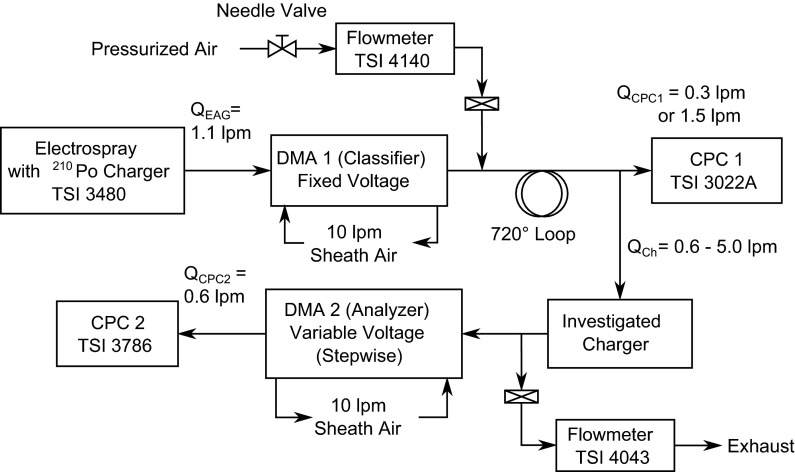
Schematic of the experimental setup for the charging probability and particle size conservation measurements

The size-dependent steady-state charging probability of the different chargers was measured by means of a tandem DMA setup with the charger of interest placed in between of the two DMAs (Fig. 1). Both of the DMAs used in this study were custom built (according to our specifications by tapcon & analysesysteme, Salzburg, Austria; Kallinger et al. 2013), hydro-mechanically identical and operating at the same polarity. In this setup, the fact was used that the particles exiting a DMA are both monomobile (i.e. of the same electrical mobility) and all of them are unipolar charged. With the investigated charger turned off, the particles remained in their state of charge, and therefore the total number-concentration at the inlet of DMA 2 could be measured with DMA 2 and CPC 2. With the charger turned on, only the number-concentration of the charged particles (with the opposite polarity to the DMAs central rod) was measured. The charging probability can be easily obtained from the ratio of the number-concentration of the charged particles to the number-concentration of the total particles.

For all steady-state charging probability measurements, it is crucially important that the charge equilibrium is achieved in the charger. If this is not the case, the calculated charging probability would be determined as too high with this setup. Since the particle charging probability was measured at different charger flow rates, the achievement of the charge equilibrium can be assumed if the results are consistent for different flow rates.

#### Experimental arrangement

Sucrose nanoparticles were produced by means of a charge reduced electrospray (Model 3480, TSI, Shoreview, USA). Depending on the needed particle size, the electrospray was operated with a 5.2 × 10^−5^–1.2 × 10^−2^ vol. conc. sucrose solution in a 20 mM ammonium acetate buffer (Chen et al. 1995; TSI Inc. 2003). A capillary of 25 µm inner diameter was used, typically with a pressure drop of 26 kPa (3.8 psi) along the 25 cm long capillary and, +1.8 kV high voltage applied on the sample which resulted in a current typically in the range of 170–240 nA. The particle-free air flow taken from a compressor was set to 1.0 lpm and mixed with 0.1 lpm CO_2_ from a high-pressure cylinder. Due to the built-in ^210^Po-charger, the aerosol exiting the electrospray generator was in charge equilibrium.

A subsequent classifier DMA (DMA 1) running at a fixed voltage was selecting a monomobile particle fraction from the particles produced by the electrospray. Due to the very narrow particle size distribution produced by the electrospray, the low charging probability for multiply charged particles in the investigated size range and an appropriate choice of the sucrose concentration the particles exiting DMA 1 were both monomobile and monodisperse.

Depending on the chosen aerosol flow rate through the charger, filtered air could be added after the classifier DMA. In case of a charger flow rate of 0.6 lpm, the excess aerosol was vented via a particle filter at this position. To ensure a uniform mixture of the aerosol with the additional air, it was routed via a 720° mixing loop into the investigated charger. CPC 1 (Model 3022A, TSI, USA) was measuring the particle concentration at the charger inlet to reduce the influence of fluctuations in particle concentration on the determination of the charging probabilities. The intake flow rate of CPC 1 was set to 0.3 lpm except for the measurements with the ^241^Am-charger at a charger flow rate of 5.0 lpm. In this case, it was set to 1.5 lpm, because otherwise the proper functioning of the CPC was influenced by the small overpressure at the inlet. The aerosol concentration at the charger inlet was kept typically between 1000 and 10,000 particles per cm^3^.

The electrical mobility distribution of the particles exiting the investigated charger was measured by means of an Analyzer DMA (DMA 2) with a variable voltage and CPC 2 (Model 3786, TSI, USA). The aerosol flow rate through DMA 2 was defined by the inlet flow rate of CPC 2 (0.6 lpm). Between the investigated charger and DMA 2, the excess aerosol was vented via a filter and a flowmeter (Model 4043, TSI, USA) for control of flow rates. In case of a charger flow rate of 0.6 lpm this port was closed. In order to change the aerosol flow rate through the charger, the flow rate was only varied in between the two DMAs. The aerosol flow rate through the DMAs was constant for all measurements. Both DMAs were operated with a sheath-air flow of 10 lpm.

All connections between the instruments where the aerosol was routed through were electrically conductive using metal connectors and electrically conductive silicone tubing (TSI, USA).

#### Measurement procedure and calculations

The charging probability measurements were done using the following procedure: the investigated charger was turned off and after a delay of 5 min 3 mobility distribution scans were performed by the analyzer DMA. Then the charger was turned on and, again after a delay of 5 min, 3 mobility distribution scans were performed. Subsequently, the procedure was repeated once, so that a measurement consists in total of 6 scans with the charger turned off and 6 scans with the charger turned on. The air flow through all chargers, except for the ^241^Am-charger, was undisturbed during measurement. In the latter case, the flow for the “off-scans” by-passed the charger.

A particle mobility distribution scan was performed by changing the applied voltage on DMA 2 in logarithmically equal steps starting at the highest voltage for the given scan and reducing it by a constant factor (16th root of 2). At every voltage step (channel) of DMA 2 particles were counted for at least 10 s by CPC 2 to ensure satisfactory statistics of counted particles after sufficient time for concentration to stabilize (~5 s).

For the determination of the charging probability at first the relative particle count (*N*_rel_) was calculated individually for each scan using:

1 $${N_{\text{rel}} = \frac{{\sum\limits_{\text{ch}} {C_{{{\text{CPC}}\, 2}} } \left( {\text{ch}} \right)}}{{C_{{{\text{CPC}}\, 1}} }}},$$

where ch denotes a channel of DMA 2 (a single voltage step), *C*_CPC2_(ch) the number of particles per second counted by CPC 2 in channel ch and *C*_CPC1_ the average particle concentration measured by CPC 1 during the whole scan. The charging probability (*P*_c_) was calculated as follows:

2 $${P_{c} = \frac{{\overline{{N_{\text{rel, On}} }} }}{{\overline{{N_{\text{rel, Off}} }} }}},$$

where the subscript On/Off denotes whether the charger was turned on or off and the over-line indicates an average of all scans (6 each). Depending on whether the singly or the doubly charged fraction was determined, the summation in Eq. 1 for the calculation o*f N*_rel, On_ was done over the distribution of the singly or the doubly charged particles in the DMA scan, respectively.

Functions like the transfer function of the DMA or the activation probability of the CPC, which need normally to be considered for the calculation of the total number-concentration from the raw data of a DMA scan (Knutson and Whitby 1975, Stolzenburg and McMurry 2008), were left out here because they would have the same contribution to *N*_rel, on_ and *N*_rel, off_ and therefore cancel out in Eq. 2. Consequently, due to the chosen measurement setup there was also no need to correct for diffusion losses. The influence of a possible non-linearity of particle counts in CPC 2, as well as space and image charge effects (inside and after the investigated charger), were assumed negligible. The finite width of the particle size distribution after DMA 1 was neglected. The particles were considered as monodisperse for the calculation of the charging probability, which practically was the case.

The measured particle mobility distributions were also used to determine the influence of the charging process on the measured particle size. This was done by a comparison of the mean measured particle size with the charger turned on and off. For that purpose the mean DMA voltage (which is inverse proportional to the particle mobility) was determined first and was calculated as the exponent of the weighted arithmetic mean of the logarithms of the voltages (*V*_eml_).

3 $${V_{\text{eml,On}} = { \exp }\left[ {\frac{{\sum\limits_{\text{ch}} { \ln } \left( {V\left( {\text{ch}} \right)} \right) \cdot C_{{{\text{CPC}}\, 2}} \left( {\text{ch}} \right)}}{{\sum\limits_{\text{ch}} {C_{{{\text{CPC}}\, 2}} } \left( {\text{ch}} \right)}}} \right]}$$

where *V*(ch) is the voltage applied on DMA 2.

Data analysis showed that a correction using the charging probability function is necessary in this calculations. A mobility distribution with the charger turned on is basically the mobility distribution with the charger turned off multiplied with the charging probability function. The latter increases strongly with increasing particle diameter in the investigated particle size range. This, together with the fact that the particles exiting DMA 1 were not perfectly monodisperse (size distribution had a narrow but finite width) caused a small (<1 %) but noteworthy size shift of the distribution towards bigger sizes which needed to be corrected. Therefore the mean voltage of the measurements with the charger turned off was calculated as follows:

4 $${V_{\text{eml,Off}} = \exp \left[ {\frac{{\sum\limits_{\text{ch}} { \ln } \left( {V\left( {\text{ch}} \right)} \right) \cdot C_{{{\text{CPC}}\, 2}} \left( {\text{ch}} \right) \cdot P_{\text{c}} \left( {Dp\left( {\text{ch}} \right)} \right)}}{{\sum\limits_{\text{ch}} {C_{{{\text{CPC}}\, 2}} } \left( {\text{ch}} \right) \cdot P_{\text{c}} \left( {Dp\left( {\text{ch}} \right)} \right)}}} \right]}$$

where *Dp*(ch) is the selected particle size at a given channel ch. For the calculation of the charging probability at a given channel *P*_c_(*Dp*(ch)) Wiedensohler’s approximation of Fuchs’ bipolar charging theory was used (Wiedensohler 1988).

The particle size ratio (*R*_s_), given by:

5 $${R_{\text{s}} = \frac{{\overline{{Dp\left( {V_{\text{eml,On}} } \right)}} }}{{\overline{{Dp\left( {V_{\text{eml,Off}} } \right)}} }} = \frac{{Cs\left( {Dp_{\text{On}} } \right)\overline{{V_{\text{eml,On}} }} }}{{Cs\left( {Dp_{\text{Off}} } \right)\overline{{V_{\text{eml,Off}} }} }}}$$

where *Cs(Dp)* is the Cunningham slip correction factor. Equation 5 can also be approximated for small differences of *Dp* with:

6 $${R_{s} = \left( {\frac{{\overline{{V_{eml,On} }} }}{{\overline{{V_{eml,Off} }} }} - 1} \right) \cdot \psi \left( {Dp} \right) + 1}$$

where the function *ψ(Dp)* represents the influence of the slip correction (see Eq. 7 in Reischl 1991).

#### Indirect measurements

**Fig. 2:**
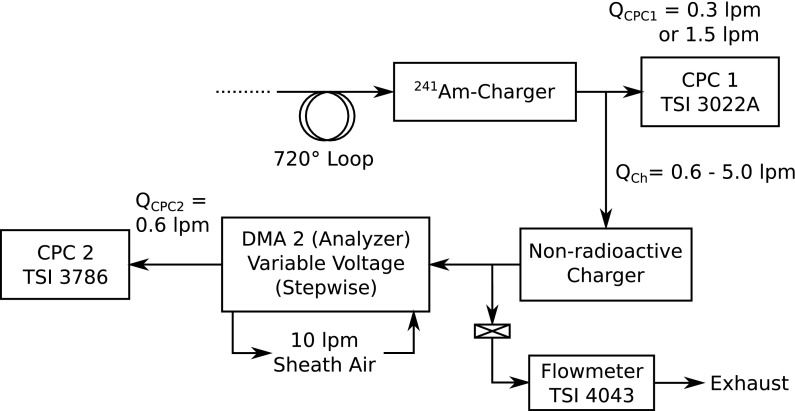
Schematic of the experimental setup for the indirect measurements

In cases when it was found that the charging probabilities measured at different flow rates were not matching, “indirect measurements” of the charging probability were performed operating two chargers in a tandem arrangement (Fig. 2). The ^241^Am-charger (in this particular setup it was only the charger and not the charger/dummy arrangement) was added directly after the 720° loop. The second charger was either the MSP EI or the TSI AAN device and was turned off and on in the same procedure as in the direct measurement.

The result of this indirect measurement is a relation of the charged particle fraction of charger 2 (one of the non-radioactive devices) to the charged particle fraction of charger 1 (the ^241^Am-charger). If the charging probability of one of the chargers is already known the charging probability of the other charger can be determined with the result of the indirect measurement.

### Relative particle penetration

**Fig. 3:**
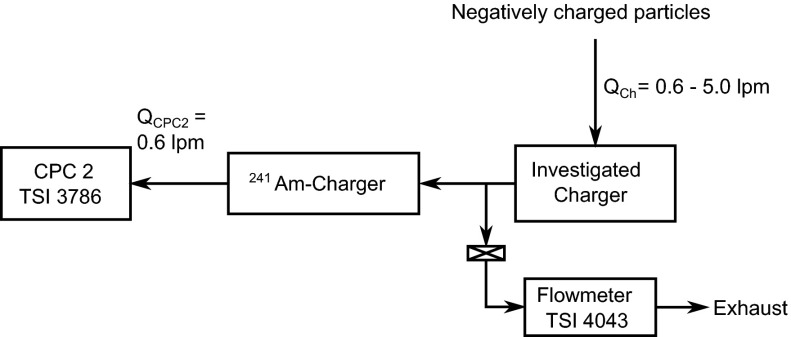
Schematic of the experimental setup for the measurement of the relative particle penetration

The particular way of the measurement of the charged particle fractions utilized in this study made it necessary to test for differences in particle penetration through the charger when it was turned on or off. The setup for this measurement (shown in Fig. 3) is similar to the setup shown in Fig. 1, except that DMA 2 was replaced by another ^241^Am-charger. This charger was placed in there to ensure that the particles entering CPC 2 were always in the same charge condition and therefore to avoid counting errors due to different activation probabilities for charged and neutral nanoparticles. The measurement procedure was also similar to the measurement of the charging probabilities, operating in an off–on–off–on cycle. The delay and measurement times were both typically set to 5 min. The measured particle counts from both CPCs were recorded every second.

## Results and discussion

All measurements reported in this paper were performed at ambient air temperature in the range of 24–26 °C and a pressure of 98–100 kPa.

### Relative particle penetration

**Fig. 4:**
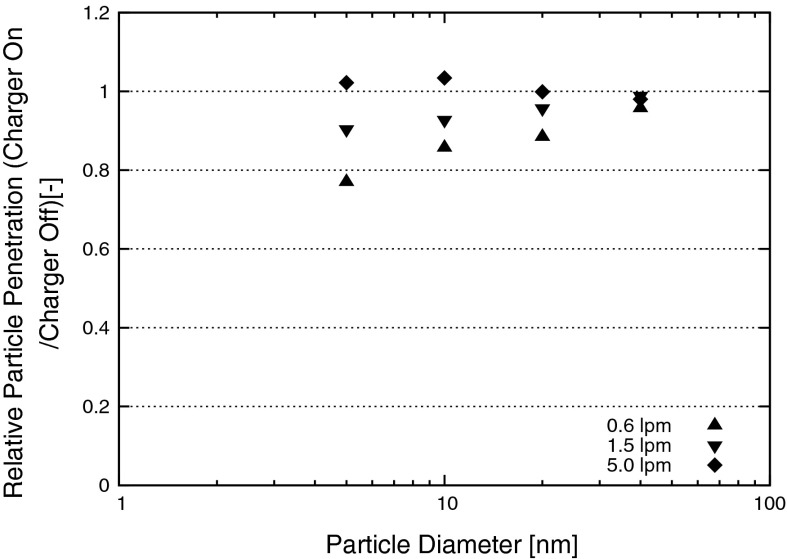
Results of the measurement of the relative particle penetration of the electrical ionizer

The relative particle penetration measurements were done at first with 5 nm particles and a flow rate of 0.6 lpm which are the smallest particle size and lowest flow rate used in this study, because the particle losses were expected to be higher with smaller particles and longer residence times inside the charger (lower flow rates). In case of the ^241^Am-charger and the soft X-ray based AAN no significant penetration differences were found. For the AC-corona based EI the relative particle penetration (charger on/charger off) was found to be 77 % (=23 % particle losses) for 5 nm and 0.6 lpm which made an investigation at other particle sizes and flow rates necessary for this device. The results of the relative particle penetration for the EI are shown in Fig. 4. Particle losses were measured to be decreasing with increasing particle size and increasing flow through the charger. Virtually no losses were measured at 5.0 lpm flow (for all particle sizes) and at 40 nm (for all flow rates). The most likely explanation for the particle losses in the EI lies in the specific way of operation of a corona charger. The corona needle produces an electrical field with high field strength. The passing aerosol particles may come in contact with this field which leads to a partial particle precipitation inside the charger.

### Charging probability

**Fig. 5:**
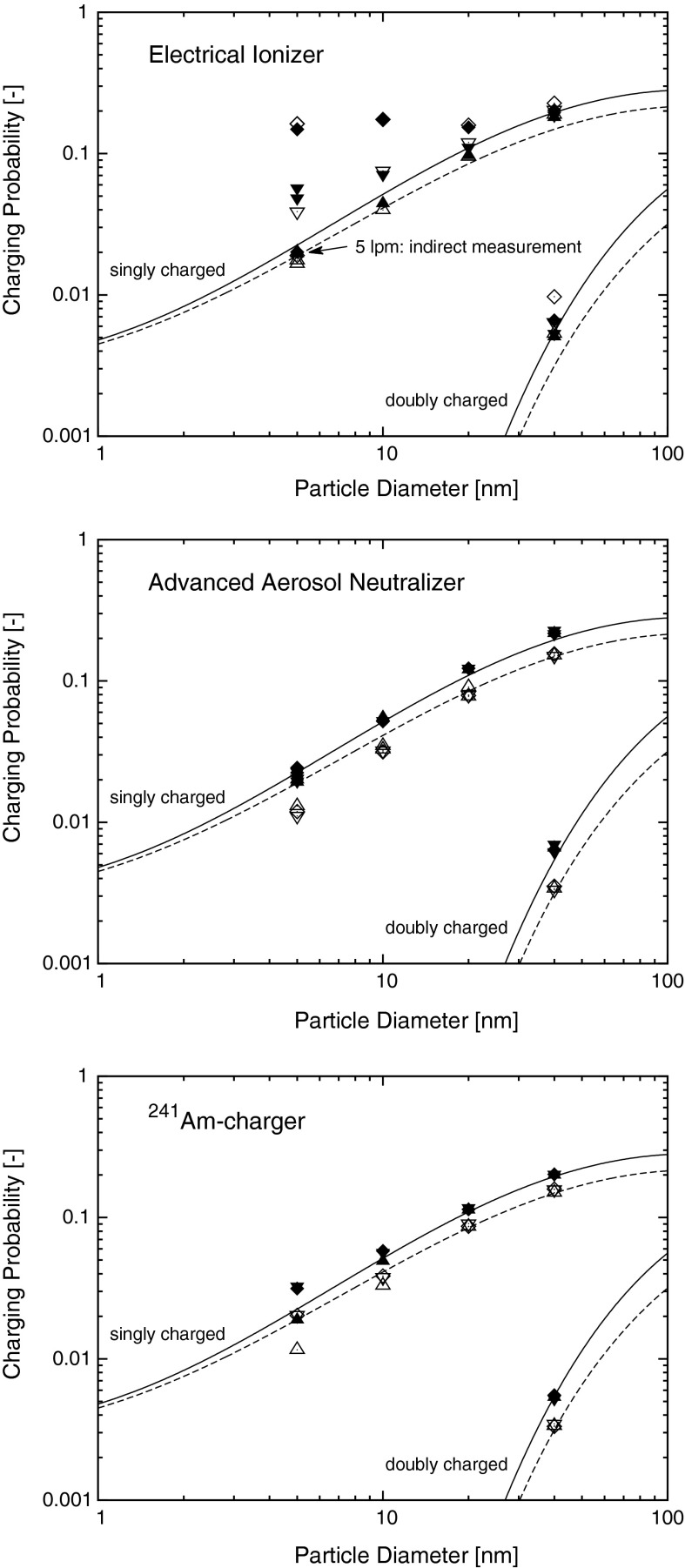
Results of the charging probability measurements of all three chargers for both polarities and three different charger flow rates. The different symbol shapes are indicating the different flow rates (*up-pointing triangle* = 0.6 lpm; *down-pointing triangle* = 1.5 lpm; *diamond* = 5.0 lpm) and the *filled* and *open style* indicates the negative and positive polarity of the particles, respectively. The *lines* represent Wiedensohler’s approximation of Fuchs’ charging theory where the *solid* and *dashed lines* stand for negative and positive polarity, respectively. *Error-bars* are within size of symbols. Values in Tables S1–S3

The results of the charging probability measurements are shown in Fig. 5. The measured values can be found in the supplementary information (Online Resource 1), Tables S1–S3.

#### Electrical ionizer

The results of the AC-corona based “Electrical Ionizer” shown in the plot are already corrected for particle penetration losses described above. The charging probabilities measured at 0.6 lpm are in an approximate agreement with the theoretical prediction (Wiedensohler 1988, with the corrected coefficients a_4_(1) and a_5_(2) from Baron and Willeke 2005), but the results for higher flow rates show a dependence with increasing flow for both polarities, which suggests that the charge equilibrium was not achieved in the charger at higher flow rates. This presumption was investigated with an indirect measurement which is discussed below.

It can be seen that the discrepancies within the different flow rates decrease with increasing particle size which may be an indication that it takes more time to bring smaller particles into charge equilibrium. This was also observed by others (Reischl et al. 1996; Qi and Kulkarni 2013) and is plausible from the charging model because the ion–particle collision probability decreases with decreasing particle size and thus decreases the ion–particle collisions per time.

A difference in the charging probability for positively and negatively charged particles as it was observed for the other two chargers and is expected because of the different physical properties of positive and negative ions (e.g. Kallinger et al. 2012) was not found with this charger. This may be caused by an unequal ion concentration ratio, due to the alternating positive and negative corona discharge.

To examine whether the charging probability results measured with the EI at high flow rates reflect the real charging probability or is because the steady-state charge equilibrium was not achieved, indirect measurements were done with 5 nm particles at 5.0 lpm flow with a tandem arrangement of the ^241^Am-charger and the EI. It has to be pointed out that this charger is specified for use for 10 nm particles and above. If the charge equilibrium is achieved in the EI the charge distribution of the exiting particles should be independent of the charge distribution of the entering particles. With the direct measurement all particles entering the EI were unipolar singly charged whereas with the indirect measurement the entering particles were already in the charge equilibrium of the ^241^Am-charger in front of the EI. Thus, if the charge equilibrium is achieved inside the EI the results from both the direct and the indirect measurement should be the same. It can clearly be seen (Fig. 5) that the results from the direct and indirect measurement differ by about one order of magnitude, so we think it is proven that for 5 nm particles the steady-state charge equilibrium when operated with a flow rate of 5.0 lpm is not fully achieved. Similarly it is assumed that the charge equilibrium was also not fully achieved for all other scenarios where the measured charging probability was increasing with a higher flow rate.

#### Advanced aerosol neutralizer

The charging probabilities measured with the soft X-ray based “Advanced Aerosol Neutralizer” show practically no dependency of the applied flow rate so it can be assumed that the charge equilibrium was achieved for all flow rates. Most of the measured data are in a good agreement with the used charging model. A notable discrepancy with the theory was measured with 5 and 10 nm positively charged particles where the measured charging probability is clearly below the theoretical prediction. Furthermore, some differences for the negatively charged particles with a diameter of 20 and 40 nm and the model are probably related to a small difference of the ion properties used in the approximation of the charging theory and of the ions produced in the charger.

#### ^241^Am-charger

The charging probabilities of the ^241^Am-charger measured with particle sizes of 20 and 40 nm show a good agreement with the theoretical prediction for all measured flow rates. However, the results for particles with diameters of ≤10 nm show a discrepancy within the measured flow rates which increases for decreasing particle size. The measurements done with a flow rate of 1.5 and 5.0 lpm deliver approximately the same result, but the charging probability at 0.6 lpm was measured to be lower. At 5 nm the difference is about a factor of 1.7. This discrepancy was found for both polarities. The theoretical prediction agrees with neither flow rate for particles ≤10 nm, but for negatively charged particles it is close to the measurement with 0.6 lpm whereas the measurements with 1.5 and 5.0 lpm are clearly above the theoretical prediction. Otherwise for positively charged particles the model is close to the points measured with 1.5 and 5.0 lpm and the measurement with 0.6 lpm derives a result well below the theory. It should be pointed out that the measured charging probabilities of the ^241^Am-charger measured with a flow rate of 0.6 lpm—although deviating from the theoretical prediction—are in an approximate agreement with the measured charging probabilities of the TSI AAN.

A possible explanation for increasing measured charging probabilities with increasing flow could be that the charge equilibrium is not achieved in the charger. But this contradicts with the fact that the measurements at both 1.5 and 5.0 lpm deliver about the same result. In the case of unachieved charge equilibrium it should be the other way around: the same result for low flow rates where the charge equilibrium is still achieved because of a longer residence time of the particles in the charger and an increasing measured charging probability with increasing flow for higher flow rates.

Because of this discrepancy an investigation with additional flow rates (0.8, 1.1 and 3.0 lpm) was done with negatively charged 5 nm particles. The results (Online Resource 1, Table S4) show an increase of the measured charging probability with increasing flow up to 1.5 lpm. At flow rates of 1.5, 3.0 and 5.0 lpm the charging probability was measured to be approximately constant. One measurement (5 nm, 0.6 lpm, negatively charged particles) was also done with the electrospray operating without CO_2_ but showed no significant change to the measurements done with typical electrospray operating conditions.

Also indirect measurements were done with the ^241^Am-charger and the AAN in tandem to exclude the influence of the switching between charger and dummy to be the reason for the charging probability discrepancy at different flow rates. The AAN was switched on an off during the measurement whereas the aerosol flow remained the same for the whole measurement. The indirect measurement with 0.6 lpm produced practically the same result as direct measurement under the same conditions, whereas the indirect measurement with 1.5 lpm delivered a smaller charging probability than the direct measurement with 1.5 lpm, but confirmed the trend of the direct measurements.

The determined different charging probabilities at varying charger flow rates may indicate a flow rate dependent mobility and mass distribution of the ions produced in the ^241^Am-charger. In a previous study we have already shown that the charger flow rate can influence the ion mobility spectra (Kallinger et al. 2012).

### Particle size conservation

**Fig. 6:**
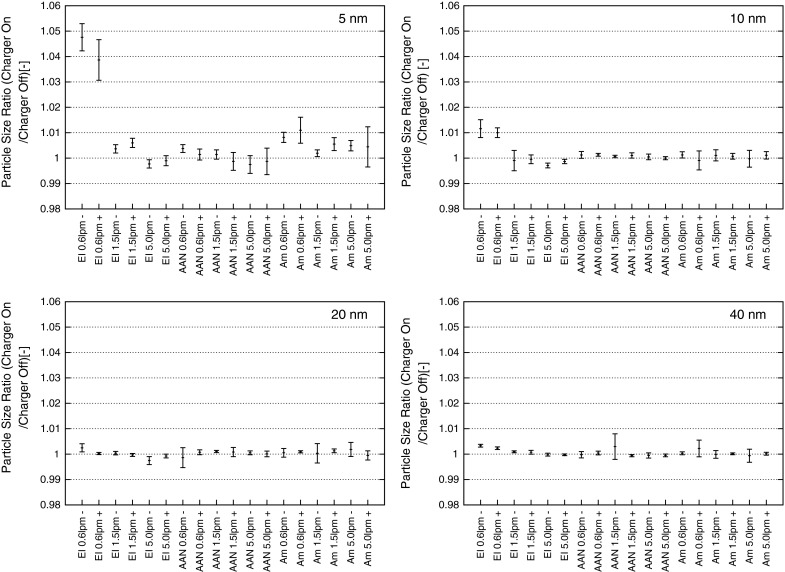
Variability of the particle size ratio for the chargers used in this study

The measured results of the particle size ratio of all chargers can be found in Fig. 6. A particle size ratio equal to one means that the particle size is conserved by the charger. The error-bars are indicating the standard deviation. Practically negligible differences in the measured particle size were determined whether the charger was on or off. A difference of about 5 % with 5 nm particles was found with the EI at 0.6 lpm flow rate. All other differences were measured to be below 1 %. The increased size (≤0.25 nm) may be an indication of an attachment on the molecular scale.

However, it has to be kept in mind that the particles entering the investigated charger had already passed a bipolar charging process due to the ^210^Po charger inside the electrospray in front of DMA 1 and were all unipolar singly charged when entering the investigated charger. It was investigated here if one of the chargers has any additional effect on the particle size of already charged particles. A possible difference of the particle size of charged and neutral particles was not investigated.

## Conclusion

The charging probabilities and the particle size conservation of two non-radioactive chargers—the MSP “electrical ionizer” (EI) and the TSI “advanced aerosol neutralizer” (AAN)—and a radioactive ^241^Am-charger was measured using airborne sucrose nanoparticles in the size range of 5–40 nm. The measurements were done at different aerosol flow rate conditions in the range of 0.6–5.0 lpm.

The AC-corona based EI produces a charge distribution approximately comparable to the model described by Wiedensohler’s approximation of Fuchs’ bipolar charging theory. However, data shows that the charger does not reach steady-state charge equilibrium at 5 lpm and, for particles with less than 20 nm in diameter, also at 1.5 lpm. Non-negligible particle precipitation for nanoparticles below 20 nm at low flow rates was determined. Therefore, the flow rate has to be deliberately chosen as a compromise between the achievement of the charge equilibrium and minimizing of particle losses when the particles of interest are in this size range. Influences on the particle size were never found to be more than 1 % for all investigated particle sizes at various flow rates, except for 5 nm particles at a charger flow rate of 0.6 lpm. It has to be stated here that the manufacturer suggests the lower particle size limit at 10 nm for this charger.

The measured charging probabilities of the soft X-ray based AAN has shown marginal differences to Wiedensohler’s approximation for particles with 20 and 40 nm in diameter. For 5 and 10 nm particles the negatively charged particles were found to be in agreement with the theory but the charging probabilities of positively charged particles were measured to be clearly below the prediction. The positive and negative charge distributions of the AAN were found be slightly more asymmetric than of a radioactive charger which stands in contradiction to previous measurements (Lee et al. 2005; Jiang et al. 2014). The measured particle size ratio was practically equal to one for the AAN.

The charging probabilities of the investigated radioactive ^241^Am-charger were found to be in agreement with Wiedensohler’s approximation for 20 and 40 nm particles. The measurements with 10 and especially 5 nm particles have shown a flow rate dependent charging probability, where the charging probabilities measured with 0.6 lpm were found to be significantly smaller than the charging probabilities measured with 1.5 and 5.0 lpm. The ^241^Am-charger has shown excellent particle size conservation.

In long term measurements, where the chargers were operated continuously for about 40 h (data not shown)—which were done with 10 nm particles at a flow rate of 1.5 lpm—no significant changes of charging probabilities were found.

## Electronic supplementary material

Supplementary material 1 (PDF 28 kb)

## References

[CR1] Adachi M, Kousaka Y, Okuyama K (1985). Unipolar and bipolar diffusion charging of ultrafine aerosol particles. J Aerosol Sci.

[CR2] Adachi M, Pui DYH, Liu BYH (1993). Aerosol charge neutralization by a corona ionizer. Aerosol Sci Technol.

[CR3] Alonso M, Kousaka Y, Nomura T, Hashimoto N, Hashimoto T (1997). Bipolar charging and neutralization of nanometer-sized aerosol particles. J Aerosol Sci.

[CR4] Baron PA, Willeke K (2005). Aerosol measurement: principles, techniques, and applications.

[CR5] Chen D-R, Pui DYH, Kaufman SL (1995). Electrospraying of conducting liquids for monodisperse aerosol generation in the 4 nm to 1.8 μm diameter range. J Aerosol Sci.

[CR6] Covert D, Wiedensohler A, Russell L (1997). Particle charging and transmission efficiencies of aerosol charge neutralizes. Aerosol Sci Technol.

[CR7] Fuchs NA (1963). On the stationary charge distribution on aerosol particles in a bipolar ionic atmosphere. Geofis Pura Appl.

[CR8] Goldman M, Goldman A, Sigmond RS (1985). The corona discharge, its properties and specific uses. Pure Appl Chem.

[CR9] He M, Dhaniyala S (2014). Experimental characterization of flowrate-dependent bipolar diffusion charging efficiencies of sub-50 nm particles. J Aerosol Sci.

[CR10] Hoppel WA, Frick GM (1986). Ion—aerosol attachment coefficients and the steady-state charge distribution on aerosols in a bipolar ion environment. Aerosol Sci Technol.

[CR11] Hussin A, Scheibel HG, Becker KH, Porstendörfer J (1983). Bipolar diffusion charging of aerosol particles—I: experimental results within the diameter range 4–30 nm. J Aerosol Sci.

[CR12] Jiang J, Kim C, Wang X, Stolzenburg MR, Kaufman SL, Qi C, Sem GJ, Sakurai H, Hama N, McMurry PH (2014). Aerosol charge fractions downstream of six bipolar chargers: effects of ion source, source activity, and flowrate. Aerosol Sci Technol.

[CR13] Kallinger P (2010) Experimentelle Untersuchung der bipolaren Diffusionsbeladung von Aerosolen mit Weichröntgenstrahlung sowie der Nanopartikelbildung unter Weichröntgenstrahlung. Master thesis, University of Vienna

[CR14] Kallinger P, Steiner G, Szymanski WW (2012). Characterization of four different bipolar charging devices for nanoparticle charge conditioning. J Nanopart Res.

[CR15] Kallinger P, Weiss VU, Lehner A, Allmaier G, Szymanski WW (2013). Analysis and handling of bio-nanoparticles and environmental nanoparticles using electrostatic aerosol mobility. Particuology.

[CR16] Kaufman SL (2010) Aerosol charge conditioner. US Patent No. US 7,796,727 B1

[CR17] Knutson EO, Whitby KT (1975). Aerosol classification by electric mobility: apparatus, theory, and applications. J Aerosol Sci.

[CR18] Kousaka Y, Adachi M, Okuyama K, Kitada N, Motouchi T (1983). Bipolar charging of ultrafine aerosol particles. Aerosol Sci Technol.

[CR19] Laschober C, Kaufman SL, Reischl G, Allmaier G, Szymanski WW (2006). Comparison between an unipolar corona charger and a polonium-based bipolar neutralizer for the analysis of nanosized particles and biopolymers. J Nanosci and Nanotechnol.

[CR20] Lee HM, Kim CS, Shimada M, Okuyama K (2005). Bipolar diffusion charging for aerosol nanoparticle measurement using a soft X-ray charger. J Aerosol Sci.

[CR21] Leong KH, Hopke PK, Stukel JJ, Wang HC (1983). Radiolytic condensation nuclei in aerosol neutralizers. J Aerosol Sci.

[CR22] Millikan RA (1913). On the elementary electrical charge and the avogadro constant. Phys Rev.

[CR23] MSP Corp. (2010) MSP product information bulletin: PI-1090, Rev. C

[CR24] Qi C, Kulkarni P (2013). Miniature dual-corona ionizer for bipolar charging of aerosol. Aerosol Sci Technol.

[CR25] Reischl GP (1991). Measurement of ambient aerosols by the differential mobility analyzer method: concepts and realization criteria for the size range between 2 and 500 nm. Aerosol Sci Technol.

[CR26] Reischl GP, Scheibel HG, Porstendörfer J (1983). The bipolar charging of aerosols: experimental results in the size range below 20-nm particle diameter. J Colloid Interface Sci.

[CR27] Reischl GP, Mäkelä JM, Karch R, Necid J (1996). Bipolar charging of ultrafine particles in the size range below 10 nm. J Aerosol Sci.

[CR28] Romay FJ, Liu BYH, Pui DYH (1994). A sonic jet corona ionizer for electrostatic discharge and aerosol neutralization. Aerosol Sci Technol.

[CR29] Shimada M, Han B, Okuyama K, Otani Y (2002). Bipolar charging of aerosol nanoparticles by a soft X-ray photoionizer. J Chem Eng Jpn.

[CR30] Steiner G, Reischl GP (2012). The effect of carrier gas contaminants on the charging probability of aerosols under bipolar charging conditions. J Aerosol Sci.

[CR31] Stolzenburg MR, McMurry PH (2008). Equations governing single and tandem DMA configurations and a new lognormal approximation to the transfer function. Aerosol Sci Technol.

[CR32] Stommel YG, Riebel U (2004). A new corona discharge-based aerosol charger for submicron particles with low initial charge. J Aerosol Sci.

[CR33] TSI Inc. (2003) Model 3480 electrospray aerosol generator. instruction manual, revision D

[CR34] TSI Inc. (2012) Model 3087 advanced aerosol neutralizer spec sheet

[CR35] Wiedensohler A (1988). An approximation of the bipolar charge distribution for particles in the submicron size range. J Aerosol Sci.

[CR36] Wiedensohler A, Fissan HJ (1991). Bipolar charge distributions of aerosol particles in high-purity argon and nitrogen. Aerosol Sci Technol.

[CR37] Wiedensohler A, Lütkemeier E, Feldpausch M, Helsper C (1986). Investigation of the bipolar charge distribution at various gas conditions. J Aerosol Sci.

[CR38] Yoon YH, Bong C, Kim DS (2015). Evaluation of the performance of a soft X-ray charger for the bipolar charging of nanoparticles. Particuology.

[CR39] Yun KM, Lee SY, Iskandar F, Okuyama K, Tajima N (2009). Effect of X-ray energy and ionization time on the charging performance and nanoparticle formation of a soft X-ray photoionization charger. Adv Powder Technol.

[CR40] Zamorani E, Ottobrini G (1978). Aerosol particle neutralization to Boltzmann’s equilibrium by AC-corona discharge. J Aerosol Sci.

