# Supplementary material for: Experimental determination of the steady-state charging probabilities and particle size conservation in non-radioactive and radioactive bipolar aerosol chargers in the size range of 5–40 nm
Source: J Nanopart Res. 2015 Apr 5;17(4):171. doi: 10.1007/s11051-015-2981-x (PMC4385563; doi:10.1007/s11051-015-2981-x)
Supplement: Supplementary file 1 — Supplementary material 1 (PDF 28 kb) [file 11051_2015_2981_MOESM1_ESM.pdf]

Journal of Nanoparticle Research

Supplemental Information:

Experimental determination of the steady-state charging probabilities and particle size conservation in non-radioactive and radioactive bipolar aerosol chargers in the size range of 5 – 40 nm

Peter Kallinger* and Wladyslaw W. Szymanski

Faculty of Physics, University of Vienna, Boltzmanngasse 5, 1090 Vienna, Austria

*Corresponding author:
Email: peter.kallinger@univie.ac.at
Tel.: +43-1-4277-73402

Table S1: Measured charging probabilities of the Electrical Ionizer with correction for particle losses. Please note that the charge equilibrium was not achieved in every measurement

Charge	Diameter [nm]	Flow [lpm]	Charging probability	Standard deviation
-1	5	0.6	0.0203	0.0013
-1	5	0.6	0.0195	0.0015
-1	5	1.5	0.0485	0.0027
-1	5	1.5	0.0571	0.0020
-1	5	5.0	0.1485	0.0118
-1	10	0.6	0.0443	0.0018
-1	10	1.5	0.0713	0.0030
-1	10	5.0	0.1752	0.0033
-1	20	0.6	0.0970	0.0062
-1	20	1.5	0.1106	0.0014
-1	20	5.0	0.1533	0.0024
-1	40	0.6	0.1805	0.0185
-1	40	1.5	0.1864	0.0051
-1	40	5.0	0.2027	0.0045
-2	40	0.6	0.0051	0.0005
-2	40	1.5	0.0053	0.0001
-2	40	5.0	0.0066	0.0003
+1	5	0.6	0.0166	0.0011
+1	5	0.6	0.0176	0.0006
+1	5	1.5	0.0388	0.0019
+1	5	5.0	0.1624	0.0138
+1	10	0.6	0.0399	0.0014
+1	10	1.5	0.0755	0.0016
+1	10	5.0	0.1745	0.0035
+1	20	0.6	0.0951	0.0021
+1	20	0.6	0.0953	0.0032
+1	20	1.5	0.1199	0.0041
+1	20	5.0	0.1587	0.0034
+1	40	0.6	0.1890	0.0060
+1	40	1.5	0.2039	0.0069
+1	40	5.0	0.2274	0.0035
+2	40	0.6	0.0053	0.0003
+2	40	1.5	0.0064	0.0003
+2	40	5.0	0.0097	0.0005

Table S2: Measured charging probabilities of the Advanced Aerosol Neutralizer

Charge	Diameter [nm]	Flow [lpm]	Charging probability	Standard deviation
-1	5	0.6	0.0201	0.0010
-1	5	0.6	0.0199	0.0011
-1	5	0.6	0.0192	0.0008
-1	5	1.5	0.0227	0.0013
-1	5	1.5	0.0205	0.0004
-1	5	1.5	0.0204	0.0012
-1	5	1.5	0.0196	0.0006
-1	5	5.0	0.0244	0.0012
-1	5	5.0	0.0224	0.0007
-1	10	0.6	0.0553	0.0010
-1	10	1.5	0.0524	0.0025
-1	10	5.0	0.0517	0.0018
-1	20	0.6	0.1197	0.0042
-1	20	1.5	0.1231	0.0020
-1	20	5.0	0.1228	0.0042
-1	40	0.6	0.2170	0.0072
-1	40	1.5	0.2162	0.0086
-1	40	5.0	0.2168	0.0044
-2	40	0.6	0.0066	0.0003
-2	40	1.5	0.0062	0.0003
-2	40	5.0	0.0064	0.0003
+1	5	0.6	0.0130	0.0008
+1	5	1.5	0.0111	0.0009
+1	5	5.0	0.0119	0.0007
+1	10	0.6	0.0328	0.0010
+1	10	0.6	0.0347	0.0007
+1	10	1.5	0.0321	0.0011
+1	10	5.0	0.0315	0.0010
+1	20	0.6	0.0777	0.0050
+1	20	0.6	0.0906	0.0026
+1	20	1.5	0.0795	0.0019
+1	20	5.0	0.0788	0.0013
+1	40	0.6	0.1517	0.0022
+1	40	1.5	0.1501	0.0016
+1	40	5.0	0.1556	0.0023
+2	40	0.6	0.0034	0.0001
+2	40	1.5	0.0033	0.0001
+2	40	5.0	0.0035	0.0001

Table S3: Measured charging probabilities of the ^{241}Am-charger

Charge	Diameter [nm]	Flow [lpm]	Charging probability	Standard deviation
-1	5	0.6	0.0189	0.0006
-1	5	1.5	0.0327	0.0008
-1	5	5.0	0.0315	0.0016
-1	10	0.6	0.0490	0.0014
-1	10	1.5	0.0567	0.0017
-1	10	5.0	0.0581	0.0038
-1	20	0.6	0.1130	0.0026
-1	20	1.5	0.1171	0.0070
-1	20	5.0	0.1140	0.0023
-1	40	0.6	0.1999	0.0042
-1	40	1.5	0.2019	0.0032
-1	40	5.0	0.2020	0.0078
-2	40	0.6	0.0053	0.0001
-2	40	1.5	0.0052	0.0001
-2	40	5.0	0.0055	0.0004
+1	5	0.6	0.0116	0.0011
+1	5	1.5	0.0205	0.0006
+1	5	5.0	0.0203	0.0018
+1	10	0.6	0.0330	0.0009
+1	10	1.5	0.0378	0.0015
+1	10	5.0	0.0386	0.0008
+1	20	0.6	0.0862	0.0012
+1	20	1.5	0.0907	0.0013
+1	20	5.0	0.0866	0.0021
+1	40	0.6	0.1503	0.0268
+1	40	1.5	0.1581	0.0014
+1	40	5.0	0.1589	0.0020
+2	40	0.6	0.0034	0.0005
+2	40	1.5	0.0035	0.0001
+2	40	5.0	0.0033	0.0001

Table S4: Additional measurements done with the ^{241}Am-charger to investigate the discrepancies at different flow rates

Charged	Diameter [nm]	Flow [lpm]	Charging probability	Standard deviation	Comment
-1	5	0.6	0.0201	0.0006	without CO_2
-1	5	0.6	0.0189	0.0011	indirect measurement
-1	5	0.8	0.0220	0.0008	
-1	5	1.1	0.0249	0.0007	
-1	5	1.5	0.0248	0.0015	indirect measurement
-1	5	3.0	0.0343	0.0007	
